# A latent profile analysis and network analysis of comorbidity of depression and anxiety in left-behind adolescents

**DOI:** 10.3389/fpsyg.2026.1751196

**Published:** 2026-02-16

**Authors:** Junying Wang, Ning Jia

**Affiliations:** Department of Psychology, Hebei Normal University, Shijiazhuang, Hebei, China

**Keywords:** depression, anxiety, left-behind adolescents, latent profile analysis, network analysis

## Abstract

**Background:**

Depression and anxiety are highly prevalent and often co-occurring mental health issues among adolescents, with comorbidity leading to poorer outcomes and additional challenges. Left-behind adolescents—a unique group experiencing disrupted parent–child relationships and limited social support—may face a higher risk of such comorbidity. Yet, few studies have examined the depression-anxiety network in this population.

**Objective:**

Latent profile analysis (LPA) identified subgroups with similar symptom patterns, and network analysis visualized the structure of comorbidities. Network comparison tests evaluated differences across subgroups.

**Methods:**

Based on the “Science Database of People Mental Health” managed by the National Population Health Data Center (China), a total of 3,205 left-behind adolescents (1,538 males; 1,667 females) were included. The Patient Health Questionnaire-9 and the Generalized Anxiety Disorder Scale-7 were used to assess depression and anxiety among left-behind adolescents.

**Results:**

Three distinct profiles were identified: high-comorbidity (8.2%), moderate-comorbidity (28.7%), and low-comorbidity (63.1%). Network structures and global strength differed significantly between subgroups. “Restlessness” was the central bridge symptom in the high-comorbidity group, while “Nervousness” was central in the moderate- and low-comorbidity groups.

**Conclusion:**

These findings suggest tailored interventions targeting subgroup-specific bridge symptoms—such as restlessness or nervousness—may improve outcomes for left-behind adolescents with comorbid depression and anxiety.

## 1 Introduction

Since the late 1970s, major social transformations in China have driven large-scale population movements. Millions of low-income farmers migrated to urban areas for higher wages, often leaving their children behind in their hometowns (Zhang et al., 2022). This has resulted in widespread parent–child separation and the emergence of a substantial population of left-behind children (LBC). LBC are typically defined as children under 18 years old who remain in rural areas, cared for by grandparents or other relatives, while one or both parents migrate to urban centers for work for at least 6 months (Zhang et al., 2023a). Among them, left-behind adolescents (LBA) refer specifically to those aged between 11 and 18 years (Yang et al., 2023). According to estimates from China’s Seventh National Population Census, the population of LBA reached 17.24 million in 2020, accounting for 25.8% of all LBC (National Bureau of Statistics of China, United Nations Children's Fund, and United Nations Population Fund, 2023). For these adolescents, this developmental stage—already a critical period of transition involving various adaptive challenges—is further complicated by prolonged separation from their parents. The absence of parental supervision, emotional support, and effective communication contributes to a less favorable environment for their physical and psychological well-being compared to their non-left-behind peers (Ge et al., 2022). The operational definition of “left-behind adolescents” in the present study is: rural household-registered adolescents aged 11–18, with one or both parents having worked as migrant workers away from home for more than six consecutive months, and who are under the care of grandparents or other relatives.

The primary mental health challenges faced by left-behind adolescents include depression and anxiety, which are highly correlated and frequently co-occur. Studies indicate that the prevalence of depression among Chinese left-behind adolescents is 26.4% (Ding et al., 2019), while anxiety disorders affect between 12.3 and 24.4% of this population (Cheng and Sun, 2015). Although depression and anxiety are classified as distinct diagnostic categories, substantial evidence indicates a high degree of symptom overlap, referred to as “comorbidity” (Meehl, 2001). Comorbidity research has garnered significant attention across various mental disorders, such as autism spectrum disorder and psychiatric comorbidities (Barlattani et al., 2023). Compared to depression or anxiety alone, their co-occurrence is associated with more severe symptoms, greater social impairment, and higher suicide risk (Haberling et al., 2019). According to attachment theory (Bowlby, 1973), the premature absence of parents may disrupt secure caregiver–child bonds in left-behind adolescents, thereby increasing vulnerability to depression and anxiety (Brumariu and Kerns, 2010). The acceptance–rejection theory (ART) further posits that parental detachment, single-parent upbringing, or care by grandparents may expose adolescents to negative relational experiences, leading to perceived rejection by significant others. This, in turn, can result in negative self-perceptions such as low self-esteem and ultimately contribute to emotional and cognitive difficulties (Rohner et al., 2005). Nevertheless, research specifically examining the network structure of depression-anxiety comorbidity in this population remains relatively scarce.

However, as noted by Fried and Nesse (2015a), substantial heterogeneity exists in symptom presentations across individuals. Even when overall severity levels of anxiety or depression are comparable, different individuals may exhibit distinct symptom patterns and clinical profiles. Therefore, although prior research suggests that left-behind adolescents are more susceptible to depressive and anxiety symptoms compared to their non-left-behind peers (Zhao et al., 2014), it is plausible that not all left-behind adolescents experience both anxiety and depression simultaneously, and symptom manifestations may vary across different subgroups within this population. Identifying subgroups with shared symptom characteristics could facilitate the development of targeted interventions for these specific clusters (Xue et al., 2024). Latent Profile Analysis (LPA), as a person-centered analytical approach, is capable of identifying homogeneous subgroups of individuals based on a set of indicators, such as diagnostic criteria, that reflect similar response patterns (Lanza et al., 2007). This method has been widely applied in adolescent research to uncover heterogeneous subtypes of psychological and behavioral characteristics within this demographic, and to further explore associated influencing factors and tailored intervention strategies (Wang et al., 2022a; Xiao et al., 2022; Zhao et al., 2019).

According to the network theory of mental disorders, the emergence and persistence of psychological issues are essentially the result of mutual influence and reinforcement among symptoms (Borsboom, 2017). These strong interactions can form feedback loops that drive the progression of the disorder. Derived from network science, network analysis provides a mathematical framework for simultaneously modeling the complex interactions among multiple factors (Borgatti et al., 2009; Borsboom and Cramer, 2013). When applied to mental health research, this approach helps identify core symptoms (i.e., key nodes in the network) and the connections between symptoms (i.e., edges linking these nodes) (Epskamp and Fried, 2018). This offers a novel perspective for understanding why multiple psychological disorders often co-occur, a phenomenon known as comorbidity (Cramer et al., 2010). For example, by constructing a symptom network of anxiety and depression, researchers can examine at a granular level how these two conditions are specifically interrelated (Xue et al., 2024). Within such a network, identifying “bridge symptoms” is crucial—these act as links between the two disorders, revealing which anxiety symptoms are most likely to trigger depressive symptoms, and vice versa, thereby pinpointing key targets for intervention (Haslbeck and Waldorp, 2020; Xue et al., 2024). Furthermore, network analysis holds clear clinical value. Intervening on a central symptom may produce widespread positive effects across the entire symptom network (Groen et al., 2020; Lunansky et al., 2022), while targeting bridge symptoms within a comorbidity network could potentially disrupt the vicious cycle between different disorders or weaken their interconnections (Xue et al., 2024). Although network analysis has been widely used to study anxiety and depression in various populations, including adolescents (Wang et al., 2022b), disabled older adults (Zhang et al., 2023b), and elderly family caregivers (França et al., 2020), no study to date has specifically employed this approach to investigate the detailed symptom-level associations within the population of left-behind adolescents.

Therefore, this study employs LPA to explore the heterogeneity in comorbidity patterns of anxiety and depression among left-behind adolescents. Additionally, network analysis was used to identify “bridging symptoms” that may link anxiety and depression in different comorbidity patterns, offering a more nuanced understanding of comorbidity and potential mechanisms. This combined “person-centered” and “variable-centered” approach aims to provide a more nuanced understanding of comorbid presentations and their underlying mechanisms in this vulnerable group.

## 2 Methods

### 2.1 Data and sample

The data used in this study were obtained from the Science Database of People Mental Health (SDPMH) of the Population Health Data Archive (PHDA). Due to ethical restrictions, the data are available upon reasonable request. SDPMH is a comprehensive study conducted across China by the National Research Institute for Family Planning, surveying children, adolescents, adults, and occupational groups, and encompassing seven major demographic segments. The survey period spanned from March 2017 to December 2022. It follows standards of the psychological industry, expert consensus, guidelines, and regulations, and uses widely accepted international and domestic standard scales suitable for Chinese norms. Data collection involved validity and reliability evaluations, and was performed following standardized procedures for both offline and online data collection (e.g., organization, training, and management) using surveys and assessments in mental health contexts. Furthermore, in 2021, the PHDA received the CoreTrustSeal global certification for trusted data repositories. Data pertaining to depression and anxiety among left-behind adolescents were extracted for analysis. The data collection was conducted between February and March 2022 across 11 junior and senior high schools in Nanchong City, Sichuan Province. The final analytical sample comprised 3,205 valid cases, including 1,538 males (47.99%) and 1,667 females (52.01%), with ages ranging from 12 to 18 years (*M* = 14.81, SD = 1.80). Because network analysis models do not allow for missing values and to improve data quality, we applied the following inclusion criteria: (1) no missing or incomplete data, and (2) no random responses (e.g., choosing the same answer for all questions). All samples met these criteria.

### 2.2 Measures

#### 2.2.1 Depression

The Patient Health Questionnaire-9 (PHQ-9), developed by Spitzer et al. (1999), was used to assess the severity of depressive symptoms. This instrument consists of 9 items, each rated on a 4-point scale from 0 (not at all) to 3 (nearly every day). The total score is calculated by summing all item scores, with higher scores indicating greater depression severity. A total score ≥ 5 may indicate the presence of depressive mood. Specifically, scores ranging from 5 to 9 may suggest mild depression, scores from 10 to 14 may indicate moderate depression, and scores from 15 to 27 may reflect severe depression. In the present study, the Cronbach’s *α* for the PHQ-9 was 0.881.

#### 2.2.2 Anxiety

The Generalized Anxiety Disorder Scale-7 (GAD-7), developed by Spitzer et al. (2006), was employed to evaluate the severity of anxiety symptoms. The scale comprises 7 items, each scored from 0 (not at all) to 3 (nearly every day), yielding a total score range of 0–21, with higher scores indicating greater anxiety severity. According to established cut-off points (Kroenke et al., 2010), scores of 0–4 indicate minimal anxiety, 5–9 mild anxiety, 10–14 moderate anxiety, and 15–21 severe anxiety. In this study, the GAD-7 demonstrated excellent internal consistency, with a Cronbach’s α of 0.946.

## 3 Data analysis

Data analysis was conducted using SPSS 26.0 for descriptive statistics, Mplus 8.3 for latent profile analysis (LPA), and R 4.4.1 (with RStudio) for network analysis.

First, LPA using Mplus 8.3 was applied to categorize classes of left-behind adolescents based on the raw scores of the 16 items from the PHQ-9 and GAD-7. The MLR estimator was used to account for the ordinal nature and potential non-normality of the item scores. Within each profile, variances and covariances were freely estimated, while these parameters were constrained to be equal across profiles. The optimal model was selected based on fit indices and theoretical interpretability. The following fit indices were considered (Peugh and Fan, 2013): (1) information criteria (AIC, BIC, and aBIC), where lower values indicate better model fit; (2) likelihood ratio tests (LMR-LRT and BLRT), where a significant *p*-value suggests that the *k*-profile model provides a better fit than the (*k*−1)-profile model; and (3) the entropy index, where values above 0.80 indicate at least 90% classification accuracy. Beyond statistical criteria, each profile was required to comprise a substantively meaningful proportion of the sample, with a minimum of 5% considered acceptable (Yin et al., 2020). After identifying the latent profiles, independent samples *t*-tests were conducted to examine differences in individual symptom levels across the profiles. Subsequently, a chi-square test was used to analyze the distribution of gender across the profiles. Additionally, the proportion of adolescents within each profile meeting the standard cutoffs for moderate-to-severe depression (PHQ-9 ≥ 10) and anxiety (GAD-7 ≥ 10) was calculated.

Second, network analysis was carried out in RStudio 4.4.2 to construct subgroup-specific networks, identify core symptoms, and examine global and local network differences. Utilizing the “qgraph” (v1.9.8) and “bootnet” (v1.6) packages in R, network estimation and visualization were conducted. The network model was constructed using the 16 items from the PHQ-9 and GAD-7 as nodes in a Gaussian Graphical Model (GGM). In the model, edges represent partial correlation relationships between two nodes after controlling for the influence of all other nodes. The thickness of the edges reflects the strength of association, with green and red denoting positive and negative associations, respectively. To simplify the model and emphasize significant connections by removing spurious edges, the graphical least absolute shrinkage and selection operator (LASSO) technique was applied to regularize the GGM. The final model was selected based on the lowest Extended Bayesian Information Criterion (EBIC) (Friedman et al., 2011), with the EBIC hyperparameter gamma (*γ*) set at 0.5 to control false positives. Furthermore, to identify central and bridge symptoms, the “networktools” package (v1.5.2) was used to calculate the expected influence (EI) and bridge expected influence (BEI) for each node. EI represents the sum of a node’s edge weights (including negative connections) to all other nodes, serving as a centrality measure of its overall network influence (Robinaugh et al., 2016). BEI, a related measure, sums the weights of edges connecting a node to those in different communities (e.g., depression to anxiety), thus quantifying its cross-community influence (Jones et al., 2021). Edge weights represent regularized partial correlations between two variables after controlling for all others in the network (Epskamp et al., 2018). To ensure network accuracy and stability, the bootnet package was used to compute the correlation stability coefficient (CS-coefficient) for node centrality (EI) and 95% bootstrapped confidence intervals (95% Bootstrapped CIs) for edge weights (Epskamp et al., 2018). A CS-coefficient should not fall below 0.25, with values above 0.50 being preferable, while narrower 95% CIs for edge weights indicate greater estimation precision. Core symptoms within each network were identified based on the highest EI values, and bridge symptoms were identified based on the highest BEI values.

Finally, the NetworkComparisonTest (NCT) package (v2.2.2) was employed to perform permutation tests (van Borkulo et al., 2023), comparing the global and local properties of the subgroup networks. Global invariance tests examined network structure invariance—whether any specific edge weight differed significantly between networks—and global strength invariance—whether the sum of the absolute values of all edge weights, reflecting the network’s overall connectivity, differed between groups. Local invariance tests examined differences in specific node expected influence values and edge weights across the networks.

## 4 Results

### 4.1 Descriptive statistics

Descriptive statistics (see Table 1) revealed that female left-behind adolescents exhibited poorer mental health outcomes, as evidenced by significantly higher scores on both depression and anxiety measures.

**Table 1:** Results of descriptive statistics (*M* ± *SD*).

Variable		Total (*n* = 3,205)	Boys (*n* = 1,538)	Girls (*n* = 1,667)	*t*	Cohen’s *d*
Depression	PHQ1: Anhedonia	0.76 ± 0.80	0.74 ± 0.81	0.78 ± 0.80	−1.40	
	PHQ2: Sad mood	0.65 ± 0.79	0.50 ± 0.72	0.79 ± 0.83	−10.63^***^	0.78
	PHQ3: Sleep	0.54 ± 0.82	0.43 ± 0.73	0.65 ± 0.88	−7.57^***^	0.81
	PHQ4: Energy	0.70 ± 0.83	0.58 ± 0.79	0.81 ± 0.85	−8.05	
	PHQ5: Appetite	0.50 ± 0.77	0.39 ± 0.70	0.60 ± 0.81	−8.10^***^	0.76
	PHQ6: Guilt	0.64 ± 0.86	0.47 ± 0.74	0.79 ± 0.92	−10.58^***^	0.84
	PHQ7: Concentration	0.50 ± 0.80	0.42 ± 0.74	0.57 ± 0.85	−5.50^***^	0.80
	PHQ8: Motor	0.40 ± 0.73	0.34 ± 0.69	0.46 ± 0.76	−4.57^***^	0.73
	PHQ9: Suicide	0.29 ± 0.66	0.19 ± 0.55	0.38 ± 0.74	−7.98^***^	0.65
	PHQ: Total score	4.98 ± 5.07	4.06 ± 4.52	5.83 ± 5.39	−10.03^***^	4.99
Anxiety	GAD1: Nervousness	0.68 ± 0.79	0.53 ± 0.72	0.81 ± 0.84	−10.11	
	GAD2: Uncontrollable worry	0.49 ± 0.78	0.35 ± 0.68	0.61 ± 0.85	−9.64^***^	0.77
	GAD3: Excessive worry	0.57 ± 0.82	0.43 ± 0.73	0.71 ± 0.88	−9.78^***^	0.81
	GAD4: Trouble relaxing	0.55 ± 0.81	0.43 ± 0.73	0.67 ± 0.86	−8.55^***^	0.80
	GAD5: restlessness	0.41 ± 0.72	0.34 ± 0.68	0.46 ± 0.75	−4.76^***^	0.72
	GAD6: Irritability	0.61 ± 0.84	0.46 ± 0.75	0.75 ± 0.89	−9.82^***^	0.83
	GAD7: Feeling afraid	0.50 ± 0.81	0.38 ± 0.72	0.61 ± 0.87	−8.34^***^	0.80
	GAD: Total score	3.80 ± 4.85	2.92 ± 4.30	4.62 ± 5.18	−10.11^***^	4.78

*p < 0.05, **p < 0.01, ***p < 0.001.

### 4.2 Latent profiles determination

Utilizing the scores of PHQ-9 and GAD-7 items as analytical indicators, the number of profiles was incrementally adjusted from 1 to 5. The results of the latent profile analysis are presented in Table 2. As the number of profiles increased, the information criteria (AIC, BIC, and aBIC) consistently decreased, and the entropy values for models 1-profile to 5-profile all exceeded 0.8, indicating progressively improved model fit. The LMR-LRT and BLRT values for all models reached statistical significance, suggesting that each model was acceptable. However, considering both parsimony and substantive interpretability, while the 2-profile model was more parsimonious, the 3-profile model demonstrated clearer and more distinct inter-profile differences. In contrast, the subgroups in the 4-profile model and 5-profile model were poorly differentiated, with one subgroup comprising less than 5% of the sample, thus lacking practical significance (Tein et al., 2013). Consequently, a three-profile solution was selected to represent the depression-anxiety co-occurrence patterns among left-behind adolescents. The average posterior probabilities for the three profiles were 0.990, 0.973, and 0.988, indicating good classification certainty.

**Table 2:** Fit statistics for latent profile analysis.

Model	AIC	BIC	ABIC	Entropy	LMR	BLRT	Class probability (%)
1-profile	121093.13	121287.45	121185.77				1
2-profile	97070.38	97367.94	97212.24	0.960	0.007	0.000	77.0/23.0
**3-profile**	**86062.37**	**86463.15**	**86253.44**	**0.967**	**0.004**	**0.000**	**63.1/8.2/28.7**
4-profile	82822.39	83326.41	83062.68	0.964	0.002	0.000	8.3/3.6/28.2/59.9
5-profile	81139.82	81747.07	81429.32	0.930	0.002	0.000	19.5/50.9/19.1/7.2/3.3

The values in the LMR and BLRT columns are the *p* values related to LMR and BLRT in comparing fit between models; *m* is the number of free parameters; LMR, Lo–Mendell–Rubin; BLRT, Bootstrapped Likelihood Ratio Test; LMR and BLRT, which were adopted to compare the estimated model and a model with *k*−1 (*k* indicating the number of profiles) profile(s), a lower and significant *p*-value means a more optimal estimated model compared to the model with one less profile. Bold values indicate the optimal latent profile classification.

Based on the dimension scores of depression-anxiety co-occurrence among left-behind adolescents, the three identified latent profiles were designated as follows: The first profile, comprising 8.2% (*n* = 262) of the total sample and characterized by the highest scores across all depression and anxiety dimensions, was labeled the High Comorbidity Profile. The second profile, accounting for 28.7% (*n* = 923) of the sample and demonstrating intermediate scores on all dimensions, was termed the Moderate Comorbidity Profile. The third profile, representing 63.1% (*n* = 2020) of the sample and exhibiting the lowest dimension scores, was designated the Low Comorbidity Profile. The chi-square test revealed a significant difference in the distribution of gender across the identified profiles (*χ*^2^ = 100.37, *p* < 0.001), with a higher proportion of females in the high-comorbidity (72.14%) and moderate-comorbidity(60.24%) groups. Additionally, there were significant differences among the groups in the proportion of adolescents who reached the moderate to severe clinical thresholds (PHQ-9 score ≥10 and/or GAD-7 score ≥10): in the high comorbidity group, 89.3% met the criteria for depression and 91.6% for anxiety, while in the moderate group, the rates were 34.2 and 38.5%, respectively. In the low comorbidity group, only 0.20% reached the threshold for moderate depression.

**Figure 1:**
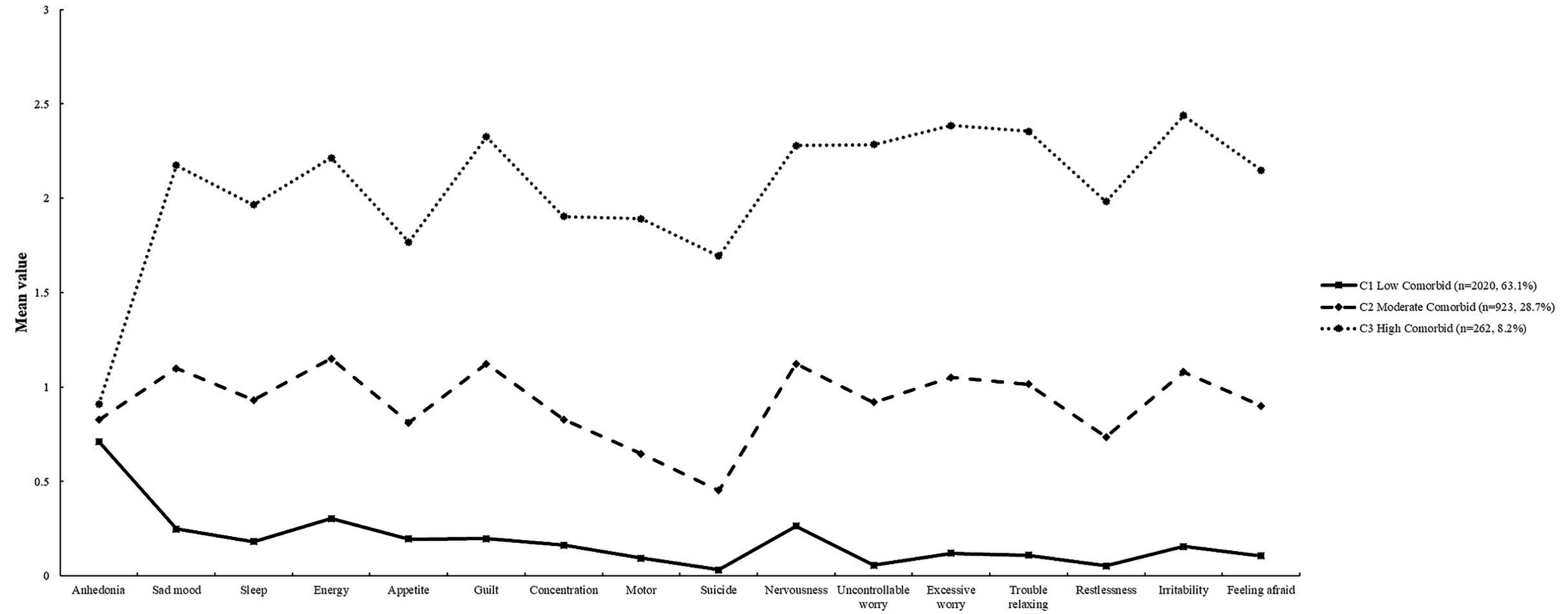
Latent profile analysis of depression-anxiety comorbid.

To examine heterogeneity across the latent profiles of depression-anxiety co-occurrence, comparisons were conducted among the three groups. The results revealed statistically significant differences in all dimension scores of depression-anxiety co-occurrence across the latent profiles (*p* < 0.001). This finding supports the validity of the latent classification of depression-anxiety co-occurrence in this population. For detailed illustrations, see Figure 1 and Table 3.

**Table 3:** Differences in depression-anxiety dimensions across latent profiles (*M* ± *SD*).

Item	C1 low comorbid	C2 moderate comorbid	C3 high comorbid	*F*	*η_p_* ^2^	*Post hoc* tests (Bonferroni)
	(*n* = 2020)	(*n* = 923)	(*n* = 262)			
PHQ1: Anhedonia	0.71 ± 0.80	0.83 ± 0.80	0.91 ± 0.85	11.39^***^	0.01	C1 < C2 < C3
PHQ2: Sad mood	0.25 ± 0.46	1.10 ± 0.57	2.18 ± 0.80	2049.48^***^	0.56	C1 < C2 < C3
PHQ3: Sleep	0.18 ± 0.44	0.93 ± 0.78	1.96 ± 0.99	1239.31^***^	0.44	C1 < C2 < C3
PHQ4: Energy	0.30 ± 0.51	1.15 ± 0.65	2.21 ± 0.80	1645.60^***^	0.51	C1 < C2 < C3
PHQ5: Appetite	0.19 ± 0.45	0.81 ± 0.76	1.76 ± 0.98	942.12^***^	0.37	C1 < C2 < C3
PHQ6: Guilt	0.20 ± 0.42	1.12 ± 0.72	2.32 ± 0.76	2192.71^***^	0.58	C1 < C2 < C3
PHQ7: Concentration	0.16 ± 0.43	0.83 ± 0.78	1.91 ± 1.04	1115.58^***^	0.41	C1 < C2 < C3
PHQ8: Motor	0.09 ± 0.33	0.65 ± 0.65	1.89 ± 1.04	1496.41^***^	0.48	C1 < C2 < C3
PHQ9: Suicide	0.03 ± 0.19	0.45 ± 0.61	1.69 ± 1.08	1500.78^***^	0.48	C1 < C2 < C3
GAD1: Nervousness	0.26 ± 0.45	1.12 ± 0.52	2.28 ± 0.73	2391.63^***^	0.60	C1 < C2 < C3
GAD2: Uncontrollable worry	0.05 ± 0.24	0.92 ± 0.58	2.28 ± 0.75	3889.27^***^	0.71	C1 < C2 < C3
GAD3: Excessive worry	0.12 ± 0.34	1.05 ± 0.61	2.39 ± 0.66	3486.29^***^	0.69	C1 < C2 < C3
GAD4: Trouble relaxing	0.11 ± 0.32	1.02 ± 0.58	2.35 ± 0.71	3537.30^***^	0.69	C1 < C2 < C3
GAD5: Restlessness	0.05 ± 0.23	0.74 ± 0.59	1.98 ± 0.90	2508.63^***^	0.61	C1 < C2 < C3
GAD6: Irritability	0.16 ± 0.39	1.08 ± 0.60	2.45 ± 0.69	3178.85^***^	0.67	C1 < C2 < C3
GAD7: Feeling afraid	0.10 ± 0.33	0.90 ± 0.72	2.15 ± 0.91	2081.90^***^	0.57	C1 < C2 < C3

***p < 0.001.

### 4.3 Network analysis

#### 4.3.1 Network estimation and accuracy

**Figure 2:**
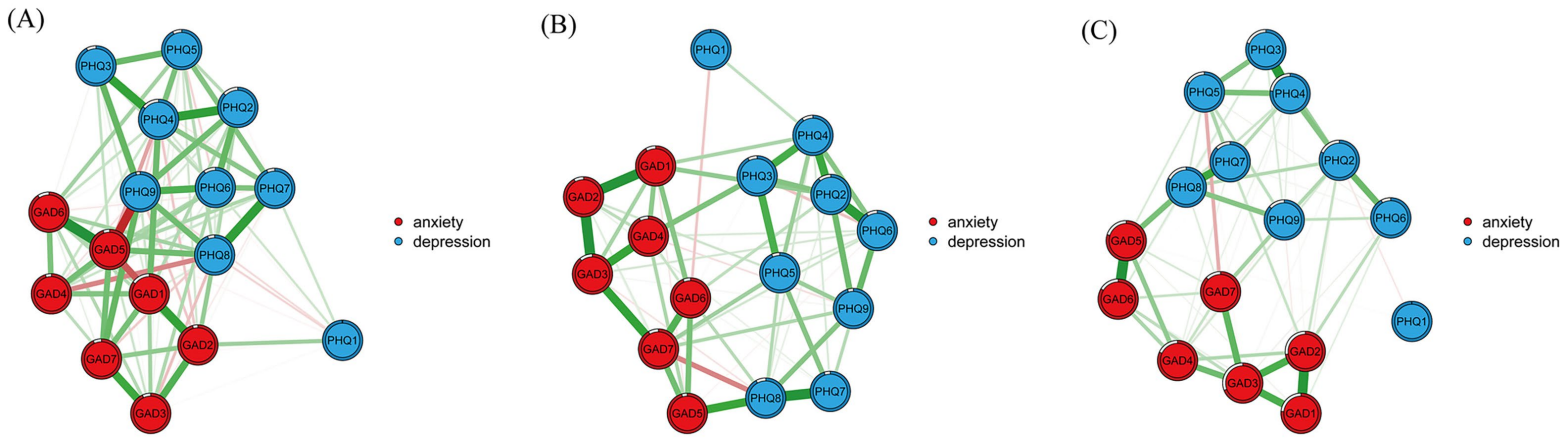
Networks of low comorbid **(A)**, moderate comorbid **(B)**, and high comorbid **(C)**. For **(A–C)**, green lines represent positive correlations, and red lines represent negative correlations. Thicker edges represent stronger associations. PHQ1, Anhedonia; PHQ2, Sad mood; PHQ3, Sleep; PHQ4, Energy; PHQ5, Appetite; PHQ6, Guilt; PHQ7, Concentration; PHQ8, Motor; PHQ9, Suicide; GAD1, Nervousness; GAD2, Uncontrollable worry; GAD3, Excessive worry; GAD4, Trouble relaxing; GAD5, Restlessness; GAD6, Irritability; GAD7, Feeling afraid.

The partial correlation networks for depression-anxiety comorbidity across the identified subgroups are presented in Figure 2. Specifically, each of the three subgroup networks comprised 16 nodes and 120 possible edges. The number of non-zero edges was 64 in the high comorbidity group, 101 in the moderate comorbidity group, and 101 in the low comorbidity group. The average edge weights were 0.41, 0.26, and 0.32 for the high, moderate, and low comorbidity groups, respectively. The assessment of network accuracy, based on the correlation stability coefficient (CS-coefficient) for node centrality and the 95% bootstrapped confidence intervals for edge weights, indicated good reliability across all subgroups. Specifically, the CS-coefficients for expected influence were 0.36, 0.44, and 0.75 for the high, moderate, and low comorbidity groups, respectively—all exceeding the recommended threshold of 0.25, thus demonstrating acceptable stability of the centrality indices. Furthermore, the relatively narrow ranges of the 95% bootstrapped confidence intervals for the edge weights suggested good precision in their estimation.

#### 4.3.2 Centrality indicators

**Figure 3:**
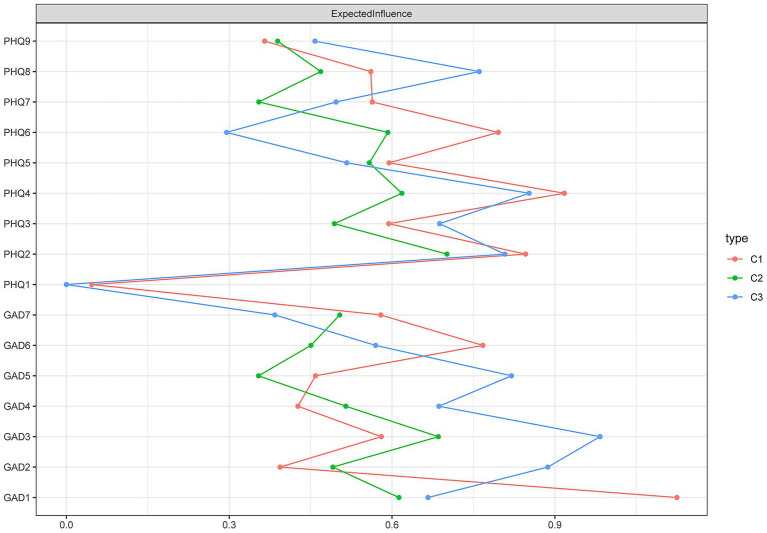
Results diagram of expected influence of low comorbid (C1), moderate comorbid (C2), and high comorbid (C3). The expected influence is defined as the sum of the values of all edges extending from a given node. The larger the value, the greater the degree of influence.

The expected influence (EI) values for each node across the different subgroups are presented in Table 4 and Figure 3. Results showed that in the high comorbidity group, the central symptoms were: GAD3 (excessive worry, EI = 0.98), GAD2 (uncontrollable worry, EI = 0.89), and PHQ4 (energy, EI = 0.85). In the moderate comorbidity group, the central symptoms were PHQ2 (sad mood, EI = 0.70), GAD3 (excessive worry, EI = 0.69), and PHQ4 (energy, EI = 0.62). For the low comorbidity group, the central symptoms were GAD1 (nervousness, EI = 1.12), PHQ4 (energy, EI = 0.92), and PHQ2 (sad mood, EI = 0.85). Notably, the symptom with the highest expected influence differed across all three subgroups.

**Table 4:** Expected influence of low comorbid, moderate comorbid, and high comorbid.

Item	Expected influence		
	C1 low comorbid	C2 moderate comorbid	C3 high comorbid
	(*n* = 2020)	(*n* = 923)	(*n* = 262)
PHQ1: Anhedonia	0.05	0.03	0.01
PHQ2: Sad mood	**0.85**	**0.70**	0.81
PHQ3: Sleep	0.59	0.49	0.69
PHQ4: Energy	**0.92**	**0.62**	**0.85**
PHQ5: Appetite	0.59	0.56	0.52
PHQ6: Guilt	0.80	0.59	0.30
PHQ7: Concentration	0.56	0.35	0.50
PHQ8: Motor	0.56	0.58	0.76
PHQ9: Suicide	0.37	0.39	0.46
GAD1: Nervousness	**1.12**	0.61	0.67
GAD2: Uncontrollable worry	0.39	0.49	**0.89**
GAD3: Excessive worry	0.58	**0.69**	**0.98**
GAD4: Trouble relaxing	0.43	0.51	0.69
GAD5: Restlessness	0.46	0.35	0.82
GAD6: Irritability	0.77	0.48	0.57
GAD7: Feeling afraid	0.58	0.61	0.38

Higher values indicate greater centrality within the network. The top three central symptoms for each network are highlighted in bold in the respective columns.

The bridge expected influence (BEI) values for each node across the different subgroups are presented in Table 5. Results indicated that the low and moderate comorbidity groups shared identical bridge symptoms, which were, in descending order: GAD1 (nervousness, BEI = 0.45), PHQ6 (guilt, BEI = 0.32), and GAD6 (irritability, BEI = 0.25). In contrast, the bridge symptoms in the high comorbidity group were GAD5 (restlessness, BEI = 0.33), PHQ8 (motor, BEI = 0.28), and GAD2 (uncontrollable worry, BEI = 0.26). Notably, the node with the highest BEI in the high comorbidity group differed from those identified in both the low and moderate comorbidity groups.

**Table 5:** Bridge expected influence (1-step) of low comorbid, moderate comorbid, and high comorbid.

Item	Bridge expected influence(1-step)		
	C1 low comorbid	C2 moderate comorbid	C3 high comorbid
	(*n* = 2020)	(*n* = 923)	(*n* = 262)
PHQ1: Anhedonia	0.10	0.10	−0.01
PHQ2: Sad mood	0.21	0.21	0.17
PHQ3: Sleep	0.13	0.13	0.05
PHQ4: Energy	0.15	0.15	0.13
PHQ5: Appetite	0.14	0.14	−0.08
PHQ6: Guilt	**0.32**	**0.32**	0.16
PHQ7: Concentration	0.06	0.06	0.12
PHQ8: Motor	0.23	0.23	**0.28**
PHQ9: Suicide	−0.12	−0.12	0.09
GAD1: Nervousness	**0.45**	**0.45**	0.03
GAD2: Uncontrollable worry	0.23	0.23	**0.26**
GAD3: Excessive worry	0.03	0.03	0.03
GAD4: Trouble relaxing	0.06	0.06	0.10
GAD5: Restlessness	0.07	0.07	**0.33**
GAD6: Irritability	**0.25**	**0.25**	0.08
GAD7: Feeling afraid	0.13	0.13	0.07

Positive values indicate a symptom acts as a bridge connecting the depression and anxiety communities. The top three bridge symptoms for each network are highlighted in bold in the respective columns.

#### 4.3.3 Network comparison

Global invariance tests were conducted to examine the overall differences among the high, moderate, and low comorbidity networks. The network structure invariance test revealed significant differences between the high and low comorbidity networks (*M* = 0.32, *p* < 0.05) and between the high and moderate comorbidity networks (*M* = 0.31, *p* < 0.05). However, no significant structural difference was found between the low and moderate comorbidity networks (*M* = 0.13, *p* = 0.54). The global strength invariance test indicated significant differences in overall connectivity between the low and moderate comorbidity networks (*S* = 0.77, *p* < 0.001) and between the moderate and high comorbidity networks (*S* = 0.90, *p* < 0.05), but not between the low and high comorbidity networks (*S* = 0.13, *p* = 0.50). The global strength values were 4.94 for the high, 4.04 for the moderate, and 4.81 for the low comorbidity group.

Local invariance tests were performed to compare differences in node expected influence and edge weights across the subgroups. The centrality invariance test showed significant differences in node expected influence among the subgroups. Overall, the high comorbidity group demonstrated stronger expected influence for its central symptoms compared to the other groups. Specifically, the expected influence for “excessive worry” (GAD3) was significantly higher in the high comorbidity group than in both the low (*p* < 0.001) and moderate (*p* < 0.001) comorbidity groups. Similarly, “uncontrollable worry” (GAD2) showed significantly higher expected influence in the high comorbidity group compared to the low (*p* < 0.001) and moderate (*p* < 0.001) groups. Furthermore, “energy” (PHQ4) in the high comorbidity group had significantly higher expected influence than in the moderate comorbidity group (*p* < 0.05).

The edge weight invariance test revealed significant differences in specific edge weights between the subgroups. Specifically, four edges differed significantly between the low and moderate comorbidity groups, two edges between the low and high comorbidity groups, and three edges between the moderate and high comorbidity groups. Further analysis showed that the following positive associations were significantly stronger in the high comorbidity group compared to both the low and moderate comorbidity groups (all *p* < 0.05): PHQ3–PHQ4, PHQ7–PHQ8, GAD1–GAD2, GAD1–GAD3, GAD2–GAD3, and GAD5–GAD6.

## 5 Discussion

This study systematically examined the heterogeneity in co-occurring depression and anxiety patterns among left-behind adolescents using latent profile analysis and network analysis. By identifying central symptoms and bridge symptoms connecting anxiety and depression across different comorbidity profiles, our findings provide a more nuanced understanding of co-occurrence patterns and their underlying mechanisms.

### 5.1 Latent profile analysis

The present study revealed distinct classification characteristics in the depression-anxiety co-occurrence patterns among left-behind adolescents. The three identified profiles demonstrated good model fit across indices, with statistically significant differences in depression and anxiety scores across profiles, indicating substantial heterogeneity in co-occurrence patterns within this population. The profiles identified in this study primarily reflect differences in symptom severity rather than qualitative distinctions in morphology. This suggests that in clinical practice, implementing stratified interventions based on severity levels may be more practical. Specifically, the depression-anxiety co-occurrence patterns were classified into three latent profiles: the high comorbidity profile (8.2%), the moderate comorbidity profile (28.7%), and the low comorbidity profile (63.1%). Adolescents in the low comorbidity profile exhibited the mildest levels of depressive and anxiety symptoms, suggesting that the majority (nearly two-thirds) of left-behind adolescents maintain relatively good mental health and demonstrate adequate adaptation to parental absence. The moderate comorbidity profile displayed intermediate symptom severity, requiring ongoing monitoring of psychological status while remaining within manageable ranges. In contrast, the high comorbidity profile showed the most severe clinical presentation, with significantly higher depression and anxiety scores than the other two profiles, representing a high-risk subgroup requiring urgent psychological intervention. These findings underscore the necessity of developing tailored intervention strategies that address the specific characteristics of each subgroup.

Our finding that females were overrepresented in the moderate- and high-comorbidity profiles aligns with extensive literature on the higher prevalence of internalizing disorders among adolescent girls (Wang et al., 2019; Zhou et al., 2015). This observed gender distribution provides important demographic context for our profiles and underscores that the identified high-risk subgroup is also one where female left-behind adolescents are disproportionately affected. While the current study was not designed to test gender moderation effects within networks due to sample size constraints in subgroups, this marked distribution highlights a critical area for future research to explore whether the symptom networks and central mechanisms operate differently for males and females within this vulnerable population.

### 5.2 Network analysis of depression-anxiety co-occurrence across subgroups in left-behind adolescents

Network analysis revealed that the key core symptoms of the depression-anxiety network differed across the three subgroups, with the high-comorbidity subgroup uniquely identified as having a critical bridge symptom. Further network analysis demonstrated that the high-comorbidity subgroup exhibited a distinct network structure compared to the other two subgroups. Thus, while the LPA provided a severity-based stratification, the subsequent network analysis revealed that these severity strata are characterized by meaningfully different functional architectures—particularly in the pathways linking anxiety and depression. This distinction helps explain the clinical complexity of the high comorbidity profile.

In the high comorbidity subgroup, generalized worry (GAD3) and uncontrollable worry (GAD2) emerged as the nodes with the strongest expected influence, suggesting that a pervasive and uncontrollable worry pattern may serve as a core driver sustaining the severe comorbidity network (Li et al., 2025). The moderate comorbidity subgroup was characterized by depressed mood (PHQ2) as its central symptom, indicating a clinical presentation more aligned with typical depressive features at this severity level. In contrast, the low comorbidity profile primarily featured feeling nervous (GAD1), highlighting the prominence of anxiety characteristics in milder co-occurrence presentations. These subgroup-specific core symptoms indicate that the functional structure of comorbid depression and anxiety varies with severity, and such differences tend to be obscured in overall network analysis (Fried and Nesse, 2015b).

However, identical bridge symptoms—specifically GAD1 (feeling nervous)—were identified in both the low and moderate comorbidity subgroups, whereas GAD5 (restlessness) was the distinctive key bridge symptom in the high comorbidity subgroup. The role of GAD1 (feeling nervous) in maintaining connections between anxiety and depressive symptoms has been consistently demonstrated across diverse populations, including secondary school students (Wang et al., 2022a, 2022b), nurses (Peng et al., 2022), and widowed older adults (Xue et al., 2024). This consistency suggests that GAD1 represents a stable bridge symptom within anxiety-depression networks, demonstrating transpopulation generalizability. According to attachment theory (Bowlby, 1973), left-behind adolescents experience impaired secure attachment due to prolonged separation from their parents, and their emotional distress often initially manifests as “nervousness.” This symptom may activate cognitive patterns such as rejection sensitivity or low self-worth, subsequently triggering depressive emotions and serving as a critical pathway in the transition from anxiety to depression.

In contrast, the high comorbidity profile was uniquely characterized by GAD5 (restlessness) as a bridge symptom, suggesting a progression to a more complex psychopathological stage. As an anxiety-related somatic symptom, the bridging role of restlessness indicates that in high comorbidity states, the interaction between anxiety and depression extends from the “emotional-cognitive” level to a multidimensional “somatic-emotional” cycle (Cramer et al., 2010). This may suggest that long-term attachment insecurity and lack of social support have led to widespread dysregulation of the emotional regulation system in left-behind adolescents. For left-behind adolescents, chronic parent–child separation and inadequate social support contribute to severe emotional distress, which becomes somatized into symptoms like restlessness. These somatic manifestations, in turn, reinforce negative emotional experiences (e.g., fatigue and psychomotor retardation associated with depression), thereby further consolidating the stability of the comorbidity network. This distinction helps explain the clinical complexity of the high comorbidity profile: compared to the low/moderate comorbidity groups, symptoms in the high comorbidity profile are more persistent and carry a poorer prognosis (Haberling et al., 2019), largely due to the establishment of somatic bridge symptoms that create self-perpetuating, cross-disorder cycles which are particularly challenging to treat.

### 5.3 Implications for practice

Current findings offer several practical implications for interventions targeting left-behind adolescents. First, employing a person-centered approach, this study identified three distinct comorbidity patterns of depression and anxiety within this population. Given the common characteristics among left-behind adolescents, such as insufficient family support and limited psychological resources, healthcare professionals can develop more targeted disease management strategies based on the symptom profiles of different subgroups. This approach enables a more rational allocation of medical resources under constrained conditions. Furthermore, network analysis revealed variations in bridge symptoms across subgroups, providing a basis for precise intervention. In the low- and moderate-comorbidity subgroups, nervousness (GAD1) serves as the core bridge symptom linking anxiety and depressive symptom clusters. Considering that left-behind adolescents often experience persistent unease due to parental separation and emotional support deficits, interventions addressing this symptom may serve as a critical entry point to prevent the progression from mild emotional distress to severe comorbidity. Therefore, psychological adaptation focusing on nervousness should constitute a core module in intervention programs for these subgroups. In contrast, the high-comorbidity subgroup exhibits a distinct network structure, with restlessness (GAD5) identified as the central bridge symptom. This manifestation may be closely associated with the chronic stress and lack of emotional soothing resources commonly experienced by left-behind adolescents. Consequently, treatment plans for this subgroup should prioritize interventions targeting somatic symptoms such as restlessness. By blocking this core driving symptom and its cross-disorder bridging pathways, it may be possible to disrupt the self-reinforcing cycle of comorbidity, thereby enhancing the precision and effectiveness of clinical interventions.

### 5.4 Limitations and future recommendations

Several limitations of this study should be acknowledged. First, the cross-sectional design precludes any causal inferences regarding the relationships between variables. Second, although the overall sample size was substantial, the sample size for the high-comorbidity subgroup (*n* = 262) was relatively small for network estimation. While the correlation stability coefficient met the minimum threshold, estimates—particularly edge weights and their comparisons—should be interpreted with caution. Future studies with larger subgroup samples are needed to verify the stability and generalizability of the distinct network structure identified in the high-comorbidity profile. Additionally, while the sample size of this study is substantial, it was drawn from Nan Chong City, Sichuan Province, and did not employ a nationwide stratified sampling method. Therefore, the generalizability of the conclusions needs to be further validated with broader samples. Finally, all measures were based on self-report questionnaires, which are susceptible to various biases and potential misinterpretation. Future research would benefit from incorporating alternative assessment methods, such as behavioral observations or clinician-rated instruments, to supplement self-report data.

## 6 Conclusion

In summary, the findings demonstrate heterogeneous patterns of anxiety-depression co-occurrence among left-behind adolescents. Network comparisons revealed significant differences in both global structure and overall connectivity strength across subgroups, with “restlessness” identified as the core bridge symptom in the high comorbidity profile and “nervousness” serving as the central bridge symptom in both the moderate and low comorbidity profiles. This study provides a theoretical framework for developing targeted interventions in future clinical practice.

## Data Availability

The datasets presented in this study can be found in online repositories. The names of the repository/repositories and accession number(s) can be found at: https://doi.org/10.12213/11.A001U.202204.281.V1.0.

## References

[ref1] Barlattani T. D’Amelio C. Cavatassi A. De Luca D. Di Stefano R. Di Berardo A. . (2023). Autism spectrum disorders and psychiatric comorbidities: a narrative review. J. Psychopathol. 29, 3–24. doi: 10.13614/2284-0249-N281

[ref3] Borgatti S. P. Mehra A. Brass D. J. Labianca G. (2009). Network analysis in the social sciences. Science 323, 892–895. doi: 10.1126/science.1165821, 19213908

[ref4] Borsboom D. (2017). A network theory of mental disorders. World Psychiatry 16, 5–13. doi: 10.1002/wps.20375, 28127906 PMC5269502

[ref5] Borsboom D. Cramer A. O. J. (2013). Network analysis: an integrative approach to the structure of psychopathology. Annu. Rev. Clin. Psychol. 9, 91–121. doi: 10.1146/annurev-clinpsy-050212-185608, 23537483

[ref6] Bowlby J. (1973). Attachment and Loss: Vol. II. Separation: Anxiety and Anger. New York: Basic Books.

[ref7] Brumariu L. E. Kerns K. A. (2010). Parent-child attachment and internalizing symptoms in childhood and adolescence: a review of empirical findings and future directions. Dev. Psychopathol. 22, 177–203. doi: 10.1017/S0954579409990344, 20102655

[ref8] Cheng J. Sun Y. H. (2015). Depression and anxiety among left-behind children in China: a systematic review. Child Care Health Dev. 41, 515–523. doi: 10.1111/cch.12221 25495395

[ref10] Cramer A. O. Waldorp L. J. van der Maas H. L. Borsboom D. (2010). Comorbidity: a network perspective. Behav. Brain Sci. 33, 137–193. doi: 10.1017/S0140525X09991567, 20584369

[ref11] Ding L. Yuen L. W. Buhs E. S. Newman I. M. (2019). Depression among Chinese left-behind children: a systematic review and meta-analysis. Child Care Health Dev. 45, 189–197. doi: 10.1111/cch.12642 30690770

[ref12] Epskamp S. Borsboom D. Fried E. I. (2018). Estimating psychological networks and their accuracy: a tutorial paper. Behav. Res. Methods 50, 195–212. doi: 10.3758/s13428-017-0862-1, 28342071 PMC5809547

[ref13] Epskamp S. Fried E. I. (2018). A tutorial on regularized partial correlation networks. Psychol. Methods 23, 617–634. doi: 10.1037/met0000167, 29595293

[ref14] França A. B. Gordon A. L. Samra R. Duarte E. S. R. Jacinto A. F. (2020). Symptoms of mood disorders in family carers of older people with dementia who experience caregiver burden: a network approach. Age Ageing 49, 628–633. doi: 10.1093/ageing/afaa008, 32091573

[ref15] Fried E. I. Nesse R. M. (2015a). Depression is not a consistent syndrome: an investigation of unique symptom patterns in the STAR*D study. J. Affect. Disord. 172, 96–102. doi: 10.1016/j.jad.2014.10.010, 25451401 PMC4397113

[ref16] Fried E. I. Nesse R. M. (2015b). Depression sum-scores don’t add up: why analyzing specific depression symptoms is essential. BMC Med. 13:72. doi: 10.1186/s12916-015-0325-4, 25879936 PMC4386095

[ref17] Friedman J. Hastie T. Tibshirani R. Glasso: Graphical LASSO–Estimation of Gaussian Graphical Models (R Package Version 1.7) [Computer software]. Comprehensive R Archive Network (CRAN). Available online at: https://CRAN.R-project.org/package=glasso

[ref18] Ge M. Yang M. Sheng X. Zhang L. Zhang K. Zhou R. . (2022). Left-behind experience and behavior problems among adolescents: multiple mediating effects of social support and sleep quality. Psychol. Res. Behav. Manag. 15, 3599–3608. doi: 10.2147/PRBM.S385031, 36514314 PMC9741830

[ref19] Groen R. N. Ryan O. Wigman J. T. W. Riese H. Penninx B. W. J. H. Giltay E. J. . (2020). Comorbidity between depression and anxiety: assessing the role of bridge mental states in dynamic psychological networks. BMC Med. 18:308. doi: 10.1186/s12916-020-01738-z, 32988400 PMC7523307

[ref20] Haberling I. Baumgartner N. Emery S. Keller P. Strumberger M. Nalani K. . (2019). Anxious depression as a clinically relevant subtype of pediatric major depressive disorder. J. Neural Transm. 126, 1217–1230. doi: 10.1007/s00702-019-02069-x, 31456039

[ref21] Haslbeck J. M. B. Waldorp L. J. (2020). Mgm: estimating time-varying mixed graphical models in high-dimensional data. J. Stat. Softw. 93, 1–46. doi: 10.18637/jss.v093.i08

[ref22] Jones P. Ma R. F. McNally R. J. (2021). Bridge centrality: a network approach to understanding comorbidity. Multivar. Behav. Res. 56, 353–367. doi: 10.1080/00273171.2019.1614898, 31179765

[ref9004] Kroenke K. Spitzer R. L. Williams J. B. W. Löwe B. (2010). The Patient Health Questionnaire Somatic, Anxiety, and Depressive Symptom Scales: a systematic review. Gen. Hosp. Psychiatry 32, 345–359. doi: 10.1016/j.genhosppsych.2010.03.006 20633738

[ref23] Lanza S. T. Collins L. M. Lemmon D. R. Schafer J. L. (2007). PROC LCA: a SAS procedure for latent class analysis. Struct. Equ. Model. Multidiscip. J. 14, 671–694. doi: 10.1080/10705510701575602, 19953201 PMC2785099

[ref24] Li T. Zhang D. Jiang T. Che W. Zhang Y. Wan Y. . (2025). A network analysis of the depression and anxiety comorbidity: a nationwide survey among Chinese adolescents during the normalization phase of COVID-19 pandemic prevention and control. BMC Psychiatry 25:598. doi: 10.1186/s12888-025-07036-3, 40598155 PMC12211814

[ref25] Lunansky G. Naberman J. van Borkulo C. D. Chen C. Wang L. Borsboom D. (2022). Intervening on psychopathology networks: evaluating intervention targets through simulations. Methods 204, 29–37. doi: 10.1016/j.ymeth.2021.11.006, 34793976

[ref26] Meehl P. E. (2001). Comorbidity and taxometrics. Clin. Psychol. Sci. Pract. 8, 507–519. doi: 10.1093/clipsy.8.4.507

[ref27] National Bureau of Statistics of China, United Nations Children's Fund, and United Nations Population Fund (2023). The population status of Chinese children in 2020: Facts and figures. Available online at: https://www.stats.gov.cn/zs/tjwh/tjkw/tjzl/202304/t20230419_1938814.html (Accessed November 10, 2025).

[ref29] Peugh J. Fan X. T. (2013). Modeling unobserved heterogeneity using latent profile analysis: a Monte Carlo simulation. Struct. Equ. Model. 20, 616–639. doi: 10.1080/10705511.2013.824780

[ref9001] Peng P. Chen Q. Liang M. Liu Y. Chen S. Wang Y. (2022). A network analysis of anxiety and depression symptoms among Chinese nurses in the late stage of the COVID-19 pandemic. Front. Public Health 10:996386. doi: 10.3389/fpubh.2022.996386 36408014 PMC9667894

[ref30] Robinaugh D. J. Millner A. J. McNally R. J. (2016). Identifying highly influential nodes in the complicated grief network. J. Abnorm. Psychol. 125, 747–757. doi: 10.1037/abn0000181, 27505622 PMC5060093

[ref31] Rohner R. P. Khaleque A. Cournoyer D. E. (2005). Parental acceptance-rejection: theory, methods, cross-cultural evidence, and implications. Ethos 33, 299–334. doi: 10.1525/eth.2005.33.3.299

[ref32] Spitzer R. L. Kroenke K. Williams J. B. (1999). Validation and utility of a self-report version of PRIME-MD: the PHQ primary care study. JAMA 282, 1737–1744. doi: 10.1001/jama.282.18.1737, 10568646

[ref33] Spitzer R. L. Kroenke K. Williams J. B. Löwe B. (2006). A brief measure for assessing generalized anxiety disorder: the GAD-7. Arch. Intern. Med. 166, 1092–1097. doi: 10.1001/archinte.166.10.1092, 16717171

[ref9003] Tein J. Y. Coxe S. Cham H. (2013). Statistical power to detect the correct number of classes in latent profile analysis. Struct. Equ. Modeling 20, 640–657. doi: 10.1080/10705511.2013.824781 24489457 PMC3904803

[ref34] van Borkulo C. D. van Bork R. Boschloo L. Kossakowski J. J. Tio P. Schoevers R. A. . (2023). Comparing network structures on three aspects: a permutation test. Psychol. Methods 28, 1273–1285. doi: 10.1037/met0000476, 35404628

[ref35] Wang S. Hou W. Tao Y. Ma Z. Li K. Wang Y. . (2022b). Mapping network connection among symptoms of anxiety, depression, and sleep disturbance in Chinese high school students. Front. Public Health 10:1015166. doi: 10.3389/fpubh.2022.1015166, 36466464 PMC9710521

[ref37] Wang F. Wu R. Yu L. X. Peng D. (2022a). Adolescent left-behind children’s loneliness and its effect factors: based on latent profile analysis. Adv. Psychol. 12, 1596–1604. doi: 10.12677/ap.2022.125191

[ref38] Wang J. Zheng Y. Li G. Li Y. Fang Z. Abbey C. . (2019). Academic achievement and mental health of left-behind children in rural China: a causal study on parental migration. China Agric. Econ. Rev. 11, 569–582. doi: 10.1108/CAER-09-2018-0194

[ref39] Xiao Y. M. Yang Y. Feng N. N. Liu B. Y. Gan Q. H. Cui L. J. (2022). The relationship between future orientation types and maladjustment of left-behind children: an application of latent profile analysis. J. Southwest Univ. (Nat. Sci. Ed.) 44, 13–19. doi: 10.13718/j.cnki.xdzk.2022.08.002

[ref40] Xue S. Lu A. Chen W. Li J. Ke X. An Y. (2024). A latent profile analysis and network analysis of anxiety and depression symptoms in Chinese widowed elderly. J. Affect. Disord. 366, 172–180. doi: 10.1016/j.jad.2024.08.181, 39214371

[ref41] Yang S. L. Tan C. X. Li J. Zhang J. Chen Y. P. Li Y. F. . (2023). Negative life events and aggression among Chinese rural left-behind adolescents: do self-esteem and resilience mediate the relationship? BMC Psychiatry 23:167. doi: 10.1186/s12888-023-04587-1, 36922776 PMC10015683

[ref42] Yin K. Peng J. Zhang J. (2020). Latent profile analysis and its application in organizational behavior research. Adv. Psychol. Sci. 28, 1056–1070. doi: 10.3724/SP.J.1042.2020.01056

[ref43] Zhang X. Q. Hong H. L. Hou W. Liu X. (2022). Prospective study of peer victimization and depressive symptoms among left-behind children in rural China: the mediating effect of stressful life events. Child Adolesc. Psychiatry Ment. Health 16:56. doi: 10.1186/s13034-022-00485-8, 35768872 PMC9245339

[ref44] Zhang L. Roslan S. Zaremohzzabieh Z. Liu K. Tang X. Jiang Y. . (2023a). A serial mediation model of negative life events on school adjustment of left-behind adolescents in rural China: the central role of hope and gratitude. BMC Psychiatry 23:588. doi: 10.1186/s12888-023-05102-2, 37580685 PMC10426169

[ref45] Zhang P. Wang L. Zhou Q. Dong X. Guo Y. Wang P. . (2023b). A network analysis of anxiety and depression symptoms in Chinese disabled elderly. J. Affect. Disord. 333, 535–542. doi: 10.1016/j.jad.2023.04.065, 37086797

[ref46] Zhao X. Chen J. Chen M. C. Lv X. L. Jiang Y. H. Sun Y. H. (2014). Left-behind children in rural China experience higher levels of anxiety and poorer living conditions. Acta Paediatr. 103, 665–670. doi: 10.1111/apa.12602, 24527673

[ref47] Zhao J. Li Q. Wang L. Lin L. Zhang W. (2019). Latent profile analysis of left-behind adolescents' psychosocial adaptation in rural China. J. Youth Adolesc. 48, 1146–1160. doi: 10.1007/s10964-019-00989-1, 30835034

[ref48] Zhou S. Hang Y. He X. (2015). Mental health status of rural female left-behind middle school students in Sichuan Province. Chin. J. Clin. Psychol. 23, 312–317.

